# Nanoparticles Effectively Target Rapamycin Delivery to Sites of Experimental Aortic Aneurysm in Rats

**DOI:** 10.1371/journal.pone.0157813

**Published:** 2016-06-23

**Authors:** Takuro Shirasu, Hiroyuki Koyama, Yutaka Miura, Katsuyuki Hoshina, Kazunori Kataoka, Toshiaki Watanabe

**Affiliations:** 1 Division of Vascular Surgery, Department of Surgery, Graduate School of Medicine, The University of Tokyo, Tokyo, Japan; 2 Translational Research Center, The University of Tokyo Hospital, Tokyo, Japan; 3 Department of Vascular Surgery, Saitama Medical Center, Saitama Medical University, Saitama, Japan; 4 Departments of Materials Engineering and Bioengineering, Graduate School of Engineering, The University of Tokyo, Tokyo, Japan; 5 Center for Disease Biology and Integrative Medicine, Graduate School of Medicine, The University of Tokyo, Tokyo, Japan; Stellenbosch University Faculty of Medicine and Health Sciences, SOUTH AFRICA

## Abstract

Several drugs targeting the pathogenesis of aortic aneurysm have shown efficacy in model systems but not in clinical trials, potentially owing to the lack of targeted drug delivery. Here, we designed a novel drug delivery system using nanoparticles to target the disrupted aortic aneurysm micro-structure. We generated poly(ethylene glycol)-shelled nanoparticles incorporating rapamycin that exhibited uniform diameter and long-term stability. When injected intravenously into a rat model in which abdominal aortic aneurysm (AAA) had been induced by infusing elastase, labeled rapamycin nanoparticles specifically accumulated in the AAA. Microscopic analysis revealed that rapamycin nanoparticles were mainly distributed in the media and adventitia where the wall structures were damaged. Co-localization of rapamycin nanoparticles with macrophages was also noted. Rapamycin nanoparticles injected during the process of AAA formation evinced significant suppression of AAA formation and mural inflammation at 7 days after elastase infusion, as compared with rapamycin treatment alone. Correspondingly, the activities of matrix metalloproteinases and the expression of inflammatory cytokines were significantly suppressed by rapamycin nanoparticle treatment. Our findings suggest that the nanoparticle-based delivery system achieves specific delivery of rapamycin to the rat AAA and might contribute to establishing a drug therapy approach targeting aortic aneurysm.

## Introduction

Aortic aneurysm is a common and lethal disease, which spontaneously expands and carries the risk of rupture [1, 2]. The current mainstream treatments for aortic aneurysm involve invasive therapies including surgical replacement with a vascular prosthesis and endovascular implantation of stent grafts; however, few drug therapies have been applied clinically to suppress aortic aneurysm formation [3]. Accumulated evidence has demonstrated that the aneurysmal formation is associated with chronic infiltration of inflammatory cells and degradation of the extracellular matrix (ECM) in the aortic wall [4]. On the basis of this finding, several drugs against such pathogenesis have been tested in animal models of aortic aneurysm and have successfully shown significant effects toward the suppression of aneurysmal formation [5–10]. However, these promising drugs have exhibited only limited therapeutic efficacies in clinical trials [11–16], potentially because no method is available to specifically deliver the drugs to the aneurysmal wall. Specific targeting to the aneurysmal wall might allow the delivery of sufficient doses of the drug for clinical effect without adverse effects to other organs, leading to the establishment of a drug therapy mechanism for aortic aneurysm.

In the present study, we attempted to develop a specific drug delivery system targeting aortic aneurysm by utilizing nanoparticles as a drug carrier. Previous histopathological studies of aneurysmal specimens revealed considerable disruption and degeneration of the wall structure of aortic aneurysms, indicating the abundance of structural micro-defects [17–20]. We therefore considered that nanoparticles in the blood stream might infiltrate and be distributed to the aneurysmal wall via these defects, which represents a possibility for realizing a specific delivery system for aortic aneurysm. To verify the approach utilizing nanoparticles, we first incorporated rapamycin into the nanoparticle (rapamycin nanoparticle), as rapamycin was previously reported to suppress aneurysmal formation by its anti-inflammatory effects [21]. Subsequently, we systemically administered the rapamycin nanoparticles to a rat model of abdominal aortic aneurysm (AAA) and evaluated their distribution and therapeutic effects against AAA.

## Materials and Methods

### Preparation of rapamycin nanoparticles (Fig 1)

Poly(ethylene glycol)-*b*-poly(γ-benzyl L-glutamate) (PEG-*b*-PBLG; number-average molecular weight = 21,000, polydispersity = 1.04, degree of polymerization in PBLG segment = 41) was synthesized based on our developed methods [22–25]. For fluorescence analysis, PEG-*b*-PBLG was labeled by conjugating the Alexa647 succinimidyl esters (Thermo Fisher Scientific, Carlsbad, CA, USA) to the ω-amino group of the polymer. In order to prepare rapamycin nanoparticles, PEG-*b*-PBLG (25 mg) and rapamycin (25 mg) were dissolved in *N*, *N*-dimethylformamide (DMF, 1.0 mL, Wako Pure Chemical Industries, Tokyo, Japan) and stirred for 24 h. The DMF solution was then added to vigorously stirred water (49 mL). The resulting suspension was purified by dialysis with a cellophane tube (Spectra/Pro 6 membrane: MWCO, 3500, GE Healthcare, Uppsala, Sweden) against water. The purified solution was concentrated by ultrafiltration using a polyethersulfone membrane (Vivaspin 6; MWCO, 300,000, GE Healthcare, Chalfont, UK) and filtered before use. To estimate the rapamycin concentration in the obtained rapamycin nanoparticles, a 1.0 mL sample of the purified solution was freeze-dried and analyzed by ^1^H nuclear magnetic resonance (^1^H NMR) spectroscopy (solvent, *d*-DMSO; *r*.*t*.; JEOL LNM-ECS 400, JEOL, Tokyo, Japan). The diameter and polydispersity index (PDI) of the rapamycin nanoparticles were measured via a dynamic light scattering method using a Zetasizer (Malvern Instruments, Worcestershire, UK). Accordingly, the rapamycin nanoparticles were suspended in 150 mM NaCl solution (pH = 7.4) and the light scattering intensities of the suspension were measured sequentially by the Zetasizer.

**Fig 1:**
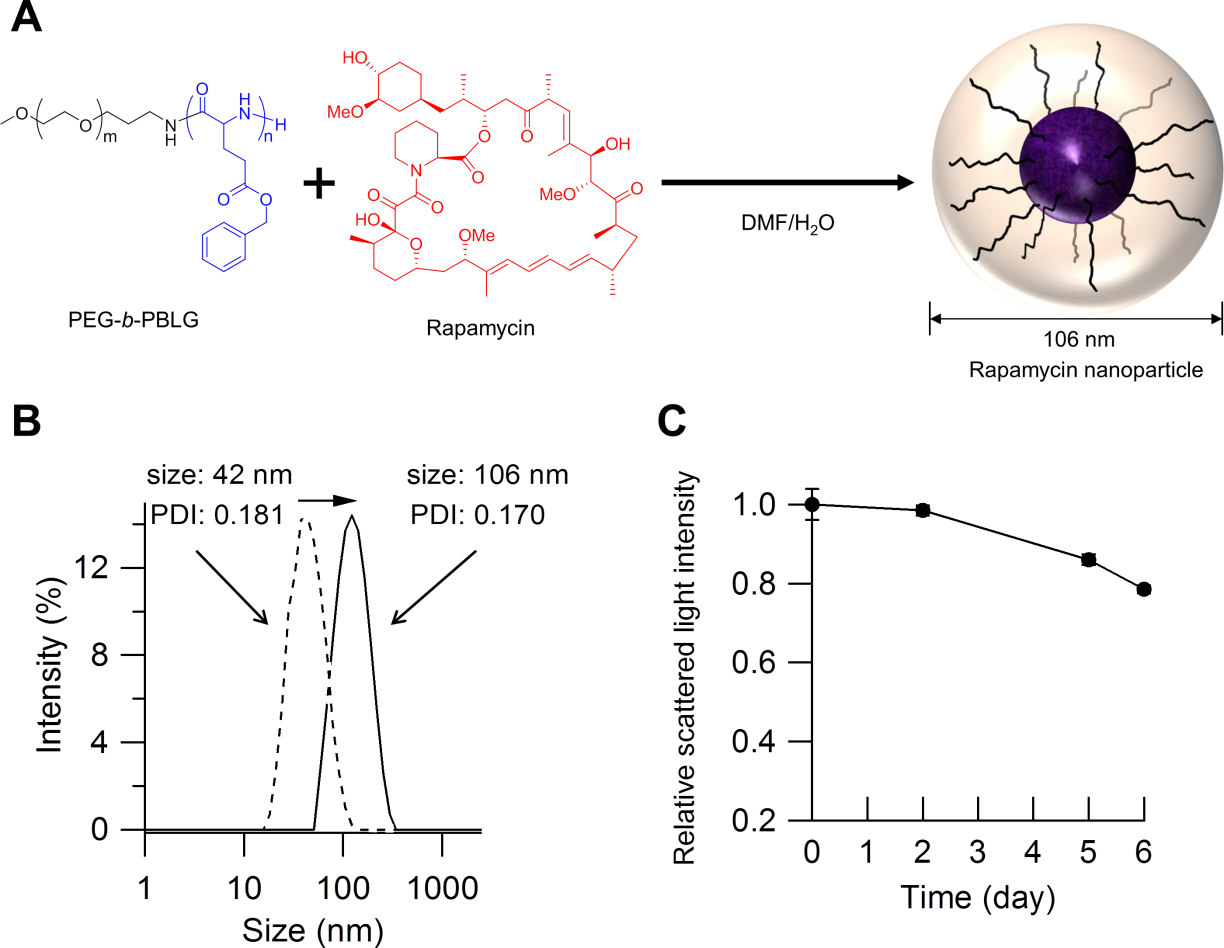
Structure and properties of rapamycin-incorporated nanoparticles. (A) Illustration showing that rapamycin-incorporated nanoparticles (rapamycin nanoparticles) are synthesized by mixing an equal mass of rapamycin and poly(ethylene glycol)-*b*-poly(γ-benzyl L-glutamate) (PEG-*b*-PBLG). Rapamycin is incorporated into the core of the PEG-shelled nanoparticle. (B) The diameter of nanoparticles measured by a Zetasizer. The size of the nanoparticles formed solely with PEG-*b*-PBLG was 42 nm (dotted line), whereas that of the rapamycin nanoparticles was 106 nm (solid line). (C) Time-course of the scattered light intensity of rapamycin nanoparticles under physiological conditions. Rapamycin nanoparticles are stable over the first 2 days. Data represent the means ± standard deviation (s.d.).

### Elastase induced model of AAA in rats

All procedures were performed as recommended by the Guide for the Care and Use of Laboratory Animals published by the US National Institutes of Health (NIH Publication, 8th edition, 2011). All protocols were approved by the Institutional Animal Care and Use Committee of the University of Tokyo (Permit Number: M-P09-017) and were performed in accordance with the institutional guidelines as stated by the University of Tokyo. Male Sprague-Dawley rats (7–8 weeks, 300–350 g; Charles River Laboratories, Yokohama, Japan), fed a normal diet, were used in the experiments. The animals were kept in an air-conditioned (21°C ± 1°C) and specific-pathogen free environment with a 12 h light-dark cycle. In an isolation rack there were 2–3 companions at most, and they had free access food and water. The total number of rats used in this experiment was 62. AAA was induced according to the method of Anidjar and Dobrin with modifications, as described [26, 27]. In brief, rats were anesthetized with inhalation of 1–2% isoflurane, with concentration adjustments if slight movement of the rats was detected. Therefore, the heart rate and respiratory rate were stable during the entire procedure. A 10-mm segment of the infrarenal aorta was completely isolated under a mid-line laparotomy. The maximum diameter of the isolated aorta was measured with electric micro-calipers in systole. Then, a PE-10 tube was introduced into the infrarenal aorta from the right saphenous artery, and the proximal and distal parts of the isolated aorta were clamped by means of temporary ligation. Through the PE-10 tube, porcine pancreatic elastase (0.27 units, Type I; Sigma-Aldrich, St. Louis, MO, USA) in 0.27 mL phosphate buffered saline (PBS) was continuously infused over 0.9 hours using a syringe pump. After the infusion, the aorta was declamped, the PE-10 tube was removed, and the maximum diameter of the isolated aorta was measured in the same manner.

### Distribution of rapamycin nanoparticles in the AAA rat model

**Fig 2:**
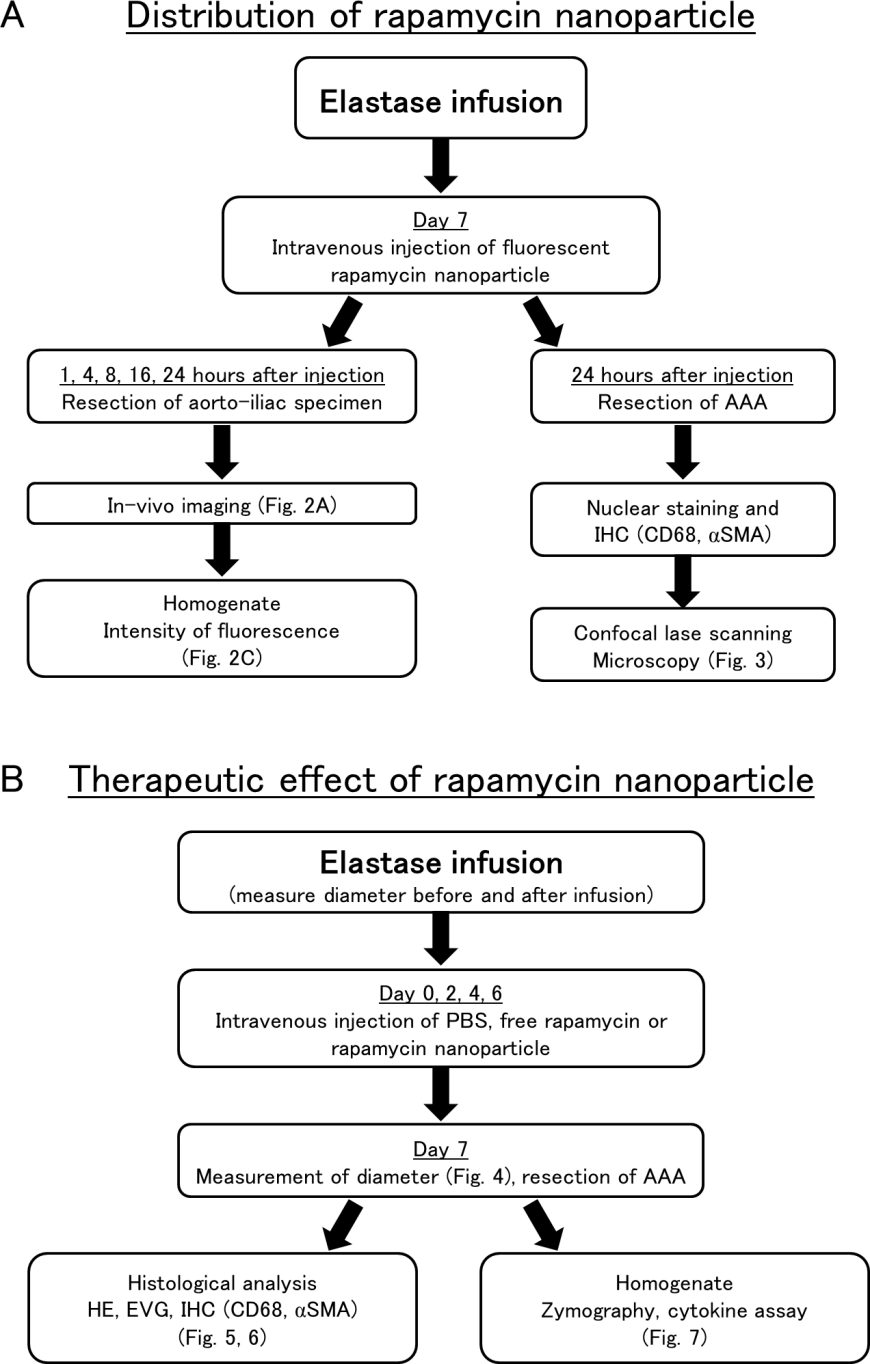
Flow-charts of the studies to analyze distribution and therapeutic effect of rapamycin nanoparticles. (A) Flow-chart of the study on the distribution of rapamycin nanoparticles to abdominal aortic aneurysm (AAA). (B) Flow-chart of the study on the therapeutic effect of rapamycin nanoparticles to suppress the formation of AAA.

Rats were subjected to elastase infusion to induce AAA, and 7 days later, 500 μg Alexa647-labeled rapamycin nanoparticles were suspended in 500 μL PBS, and the suspension was injected via the tail vein of the rats (*n* = 19, Fig 2A). After blood sampling, the animals were killed by an anesthesia overdose at 1, 4, 8, 16, or 24 hours after injection (at 1, 4, 8, 24 hours, *n* = 4; at 16 hours, *n* = 3) after blood sampling. Subsequently, the thoracic aorta, abdominal aorta, and bilateral iliac arteries were harvested together, and were gently rinsed with PBS to wash out the remaining blood. The aorto-iliac specimens were set flat with the anterior side facing upwards, and the distribution of Alexa647-labeled rapamycin nanoparticles was first evaluated with an IVIS® imaging system (excitation filter: 640 nm, emission filter: 680 nm, PerkinElmer, Hopkinton, MA, USA). As a control to eliminate the intrinsic fluorescence of the rat aortic tissue, another rat (*n* = 1) was subjected to elastase infusion and sacrificed 7 days later without injection of Alexa647-labeled rapamycin nanoparticles, followed by sample collection in the same manner.

For quantitative evaluation, plasma was separated from each blood sample, and the fluorescence intensities of the plasma samples were measured at 680 nm. The distribution half-life and elimination half-life were estimated, and the residual quantities of rapamycin nanoparticles in the plasma (residual ratio) were calculated as described previously [28]. The segment of AAA and the thoracic aorta were cut out individually from each aorto-iliac specimen after the evaluation by IVIS®. Each segment was weighed (W_sample_ [g]) and lysed in 1 mL Passive lysis buffer (E194A, Promega, Fitchburg, WI, USA). The absorbance of each lysate at 680 nm (A_sample_) was measured using an Infinite® microplate reader (Tecan, Männedorf, Switzerland). The weight and absorbance of the control sample were also measured (W_control_ [g] and A_control_). A_sample_ data were adjusted as values per 1 g sample weight as follows:

$$Adjusted\;absorbance\;value=\frac{Asample}{Wsample}+\frac{Acontrol}{Wcontrol}$$

### Histology of rapamycin nanoparticles distributed to the AAA

To analyze the microscopic distribution of rapamycin nanoparticles in the AAA wall, another set of rats (*n* = 3) were subjected to elastase infusion and treated with injection of Alexa647-labeled rapamycin nanoparticles at day 7 in the same manner. The middle segments of the AAAs were excised from each rat at 1 day after the particle injection and embedded in OCT compound (Sakura Finetek, Tokyo, Japan). Transverse cross sections (10 μm) were stained by Hoechst 33342 (Thermo Scientific, Waltham, MA, USA), and the section was analyzed by confocal laser scanning microscopy (LSM 780, Carl Zeiss, Oberkochen, Germany).

Other sections were immuno-stained for CD68 or α-smooth muscle actin (αSMA) to evaluate the co-localization of rapamycin nanoparticles with macrophages and smooth muscle cells (SMCs), respectively. After blocking (Blocking one, Nacalai Tesque, Inc., Kyoto, Japan), a monoclonal antibody against CD68 (1:100; T-3003, BMA Biomedicals, Augst, Switzerland) or against αSMA (1:100; M0851, Dako, Glostrup, Denmark) was applied, and the sections were incubated overnight at 4°C. Then, the sections were treated with Alexa Fluor® 555-labeled secondary antibody (1:300; ab150118, Abcam, Cambridge, UK) and analyzed by confocal laser scanning microscopy.

### Therapeutic effects of rapamycin nanoparticles

Rats were randomly assigned to five treatment groups, and elastase was infused into the infrarenal aorta of each animal as described. Measurement of the maximum diameter of the isolated aorta was conducted in each animal before and after elastase infusion as described above (Fig 2B). Immediately after and at 2, 4, and 6 days after the elastase infusion, the rats of each group respectively received intravenous injections of the following suspension or solution via the tail vein (i.e., injections four times in each group): i) 1 mL/kg PBS alone (PBS, *n* = 6), ii) 0.1 mg/kg free rapamycin in 1 mL/kg special solvent (free/RAP-0.1, *n* = 6), iii) 1 mg/kg free rapamycin in 1 mL/kg special solvent (free/RAP-1, *n* = 6), iv) 0.1 mg/kg rapamycin nanoparticles in 1 mL/kg PBS (RAP/nano-0.1, *n* = 6), or v) 1 mg/kg rapamycin nanoparticles in 1 mL/kg PBS (RAP/nano-1, *n* = 6). The special solvent for free rapamycin consisted of 5% Tween 80, 5% polyethylene glycol 400, and 5% ethanol in distilled water [29]. At 7 days after the elastase infusion, the induced AAA of each animal was exposed, and the maximum diameter of each AAA was measured in systole. The diameter ratio is expressed for each rat as the ratio of the diameter after elastase infusion to the diameter before elastase infusion. Subsequently, all rats were sacrificed and subjected to perfusion fixation with 4% phosphate buffered paraformaldehyde (0.1 mol L PO_4_, pH 7.3) at 120 mmHg. The segment of AAA was excised and immersed in the same fixative for 1 hour. The sample segment was excised from the middle part of the AAA and embedded in paraffin.

### Histological analyses of the AAA wall

Transverse cross sections (5 μm) of the AAA wall were cut from paraffin-embedded samples. The sections were stained by hematoxylin-eosin (HE) or Elastica van Gieson (EVG) staining. Other sections were immuno-stained for CD68 or αSMA. After hydrogen peroxide treatment, blocking, and incubation with primary antibodies as described, the sections were treated with biotin-labeled secondary antibody (1:500; sc-2039, Santa Cruz Biotechnology, Dallas, TX, USA) and visualized using an avidin-biotin complex detection kit (ABC Elite kit, Vector Laboratories, Burlingame, CA, USA). Photomicrographs were taken, and the area of the aneurysmal wall was measured in each section using Image J software (NIH, Bethesda, MD, USA). The CD68-positive cells in the aneurysmal wall were counted, and the CD68-positive cell density was calculated as follows:

$$CD68-positive\;cell\;density=\frac{number\;of\;CD68-positive\;cells}{specimen\;area}$$

### Gelatinase activity and expression of inflammatory cytokines

Another set of rats was assigned to three treatment groups, and elastase was infused into their infarenal aortas. According to the same protocol as described, the rats of each group received injections of PBS (*n* = 3), free/RAP-1 (*n* = 3), or RAP/nano-1 (*n* = 3) immediately after and at 2, 4, and 6 days after the elastase infusion. At 7 days after elastase infusion, the segment of AAA was excised from each animal and lysed in 200 μL Passive lysis buffer. Gelatinase activity of the tissue lysates was determined using a gelatin-zymography kit (AK47, Primary Cell Co, Sapporo, Japan) according to the manufacturer’s instruction. Equal total protein (50 μg) amounts of each lysate were separated on SDS-PAGE containing 1% gelatin, and the SDS-PAGE gel was incubated in incubation buffer for 10 hours at 37°C. Matrix metalloproteinase (MMP) marker (included in the gelatin-zymography kit, see supplier’s data sheet: http://search.cosmobio.co.jp/cosmo_search_p/search_gate2/docs/PMC_/AK47COS_E.20160418.pdf) containing MMP-2, the latent form MMP-2 (pro-MMP-2), and the latent form MMP-9 (pro-MMP-9) were applied in separate lanes. The gel was scanned using a transmission model scanner (ESPER-SCANNER®, Seiko Epson, Nagano, Japan) for zymography analysis, and the densitometric analysis was performed using Image J software. In addition, the expression of the inflammation-related cytokines and chemokines in the tissue lysates was evaluated using a Proteome Profiler Array kit (rat cytokine array panel A, R&D Systems, Minneapolis, MN, USA) according to the manufacturer’s instruction [30]. This kit enables the profiling of the relative levels of the following cytokines and chemokines between tissue lysates: cytokine-induced neutrophil chemoattractant (CINC)-1, CINC-2α/β, CINC-3, interleukin (IL)-1α, IL-1β, IL-6, IL-10, IP-10, macrophage inflammatory protein (MIP)-1α, MIP-3α, regulated on activation, normal T cell expressed and secreted (RANTES), and tumor necrosis factor (TNF)-α. Each tissue lysate with 400 μg total protein was applied to the array. The resultant array membrane was exposed to X-ray film and the pixel densities on the developed X-ray film were collected using a transmission model scanner. The acquired data were analyzed by Image J software.

### Statistical analyses

The values are expressed as the means ± standard deviation. Dunnett’s test was applied to determine the significance of the differences compared to the control group. The differences between two groups were analyzed by an unpaired Student’s *t*-test. A *p* value < 0.05 was considered to be significant.

## Results

### Characteristics of rapamycin nanoparticles

Rapamycin-incorporated nanoparticles were simply prepared by mixing an equal mass of rapamycin and PEG-*b*-PBLG (Fig 1A) [31]. The yield of rapamycin incorporation was measured by ^1^H NMR and estimated to be 82%. The analyses using the Zetasizer revealed that the diameter of the rapamycin nanoparticles was 106 nm with a moderate PDI (*i*.*e*., 0.170), while that of particles formed solely with PEG-*b*-PBLG was 42 nm (Fig 1B). Under physiological conditions (pH = 7.4, 150 mM NaCl, 37°C), the scattered light intensities of the rapamycin nanoparticles were stable during the initial 2 days. Thereafter, the scattered light intensities decreased gradually, although the value at day 6 remained at 78% of that at the start of measurement (Fig 1C).

### Distribution and plasma clearance of rapamycin nanoparticles

**Fig 3:**
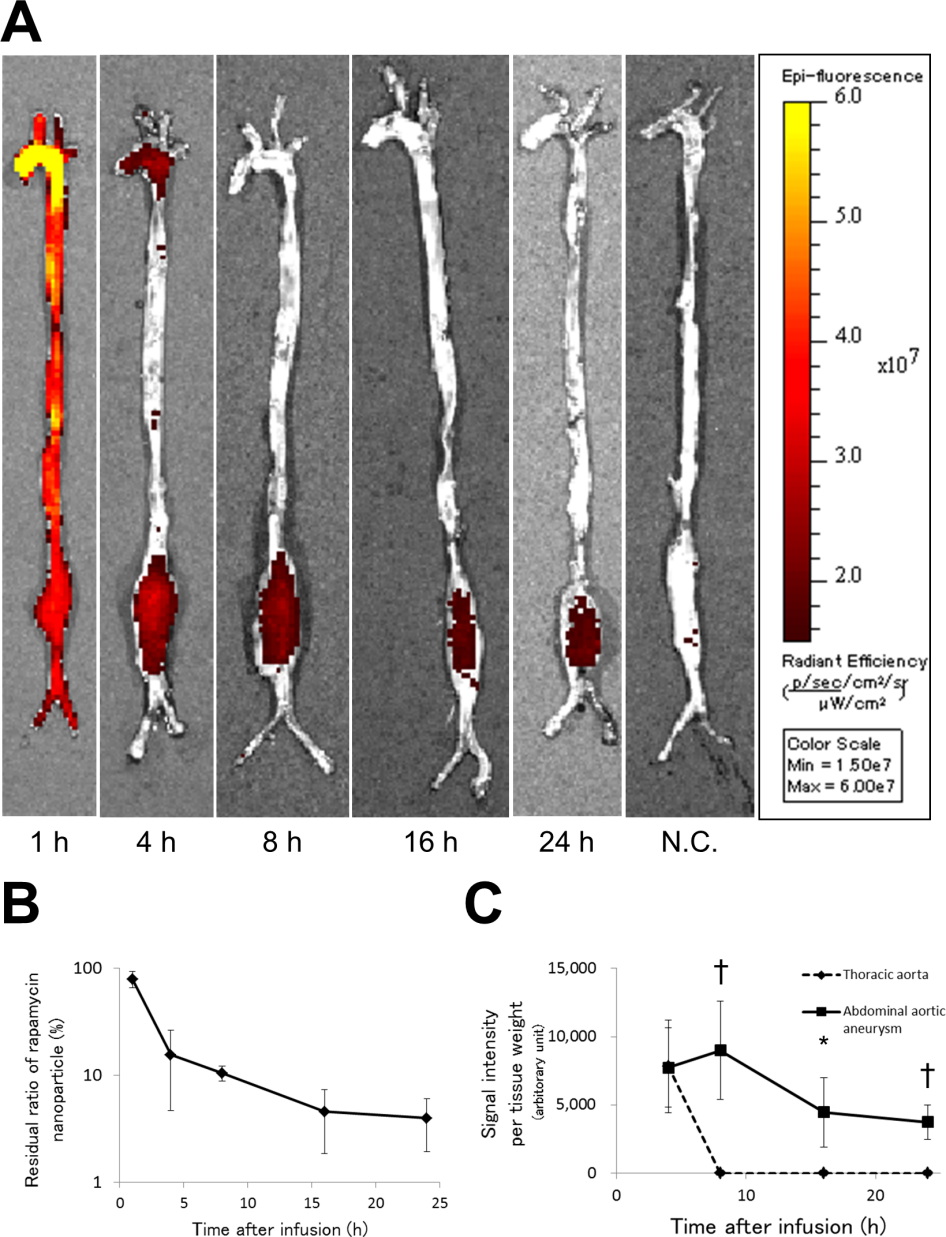
Accumulation of Alexa647-labeled rapamycin nanoparticles in the AAA rat model. (A) Representative images of macroscopic distribution of Alexa647 in the aorto-iliac specimens as imaged using the IVIS® imaging system. Abundant signals of Alexa647 were specifically distributed in the AAA at 4, 8, 16, and 24 hours after injection of Alexa647-labeled rapamycin nanoparticles (red-staining, at 1, 4, 8, and 24 hours, *n* = 4; at 16 hours, *n* = 3). (B) The plasma clearance of rapamycin nanoparticles in the rat model. The residual ratio of rapamycin nanoparticles in the plasma was high at 1 hour after injection. (C) Fluorescence intensities of the lysates of AAA and the thoracic aorta are shown as adjusted absorbance values. The values of AAA were significantly higher than those of the thoracic aorta at 8, 16, and 24 hours after injection. Data represent the means ± s.d. N.C. indicates negative control. *p < 0.05, †*p* < 0.01 (unpaired Student’s *t*-test).

To assess the *in vivo* distribution of the rapamycin nanoparticles, Alexa647-labeled rapamycin nanoparticles were intravenously injected into rats in which AAA had been induced by infusing elastase into the abdominal aorta. The animals were euthanized by overdose anesthesia at 1, 4, 8, 16, or 24 hours after injection (at 1, 4, 8, 24 hours, *n* = 4; at 16 hours, *n* = 3) after blood sampling. In the aorto-iliac specimens harvested at 1 hour after injection, IVIS® imaging showed abundant fluorescence signal over the entire specimens, and a strong intensity of fluorescence on the aortic arch as compared with other parts of the specimens (Fig 3A). At 4 hours after injection, evident fluorescence signal was detected on the segments of the aortic arch with AAA, and then the evident fluorescence signal was restricted to only the AAA segment at 8, 16, and 24 hours after injection.

Before harvesting the specimens at 1, 4, 8, 16, or 24 hours after injection, a plasma sample was collected from each animal. Then, the fluorescence intensities of the plasma samples were measured, and the clearance of the rapamycin nanoparticles was calculated. The distribution half-life and elimination half-life were estimated as 2.1 hours and 15.8 hours, respectively (Fig 3B). The residual ratio of rapamycin nanoparticles in the plasma was considerably elevated at 1 hour after the injection of Alexa647-labeled rapamycin nanoparticles, although the value decreased to 15% at 4 hours after the injection.

The segments of AAA and thoracic aorta were excised from the aorto-iliac specimens after IVIS® analyses, and the adjusted absorbance values of their lysates were calculated. In these values, we rejected the data at 1 hour after an injection, since the values at 1 hour were markedly biased by the high intensity of plasma (Fig 3C). The adjusted absorbance values of AAA exhibited a delayed peak at 8 hours after injection, whereas the values of the thoracic aorta diminished and became zero by 8 hours. The values of AAA were significantly higher than those of the thoracic aorta at 8, 16, and 24 hours after injection.

### Histology of rapamycin nanoparticles distributed to the AAA

**Fig 4:**
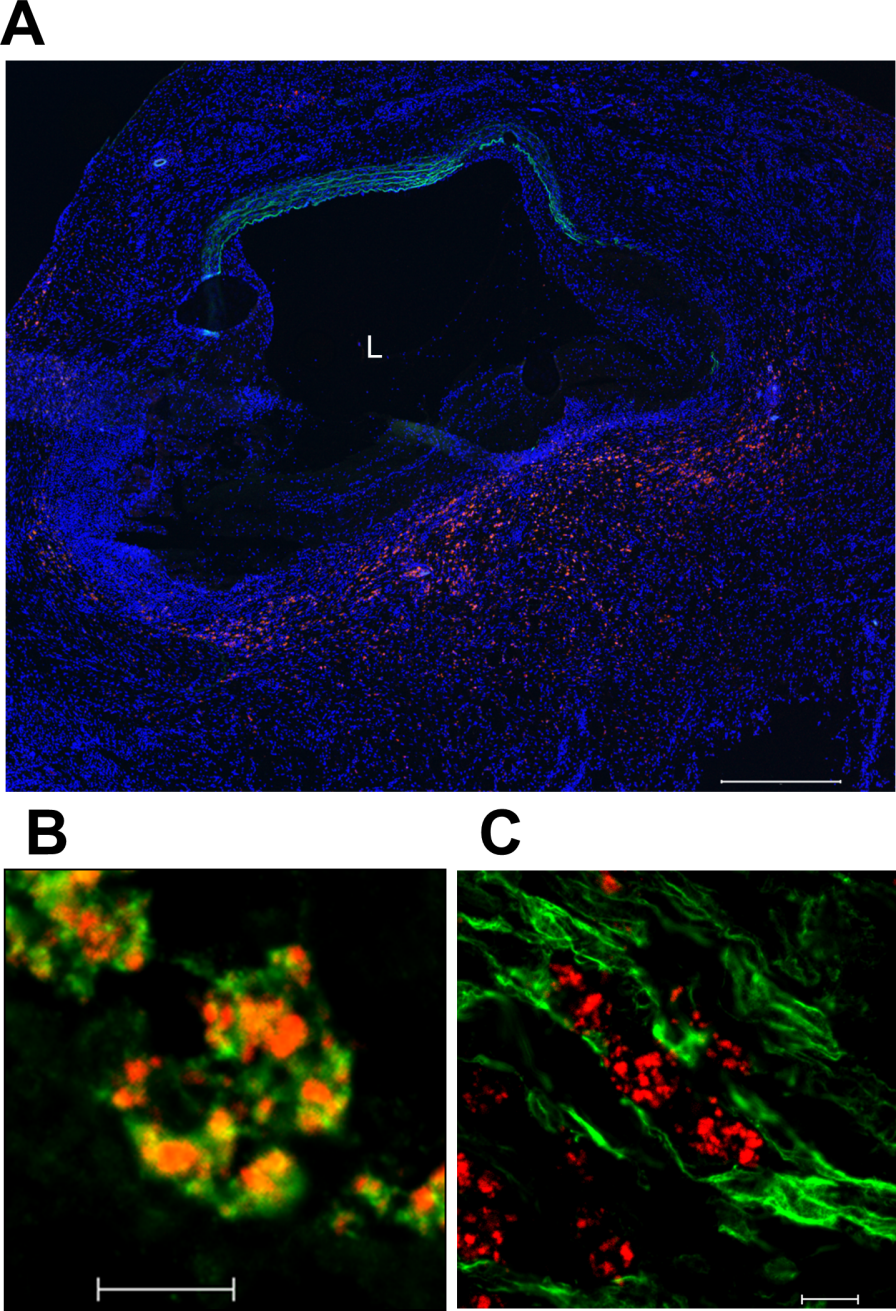
Microscopic distribution of Alexa647-labeled rapamycin nanoparticles in the rat AAA. (A) Confocal laser scanning micrograph of the rat AAA 7-days post induction and 24 hours after injection of the Alexa647-labeled rapamycin nanoparticles. Note the abundant accumulation of Alexa647-labeled rapamycin nanoparticles (red dots) distributed in the media and adventitia with progressive destruction of the wall structure. Nuclei are stained by Hoechst33342 (blue dots), and elastic laminae of the media are visualized by intrinsic fluorescence (green). L indicates the lumen, scale bar = 500 μm. (B) Micrograph of the cross sections stained for CD68 (green). Co-localization with Alexa647-labelled rapamycin nanoparticles (red dots) appears as a yellow color. The majority of nanoparticle dots were co-localized with CD68-positive cells (S1A Fig). Scale bar = 10 μm. (C) Micrograph of the cross sections stained for αSMA (green). There is little co-localization with Alexa647-labeled rapamycin nanoparticles (red dots, S1B Fig). Scale bar = 10 μm.

Alexa647-labelled rapamycin nanoparticles were injected into the rat AAA model in the same manner, and the microscopic distribution of the nanoparticles was analyzed. Confocal laser scanning microscopy detected the Alexa647-labelled rapamycin nanoparticles in the sections of AAA as small fluorescent dots, and also depicted the intrinsic fluorescence of the medial elastic laminae of the AAA wall, which provided information concerning its structural destruction. There was no other auto-fluorescence such as that of red blood cells. In all sections of the AAA samples, the small fluorescent dots were distributed to the media and adventitia with progressive destruction of the wall structure (Fig 4A). Sections immuno-stained for CD68 revealed that the majority of the small fluorescent dots were co-localized with CD68-positive cells (Fig 4B, S1 Fig). In contrast, immuno-staining for αSMA showed little co-localization of the fluorescent dots and αSMA-positive cells (Fig 4C).

### Inhibition of AAA enlargement by rapamycin nanoparticles

**Fig 5:**
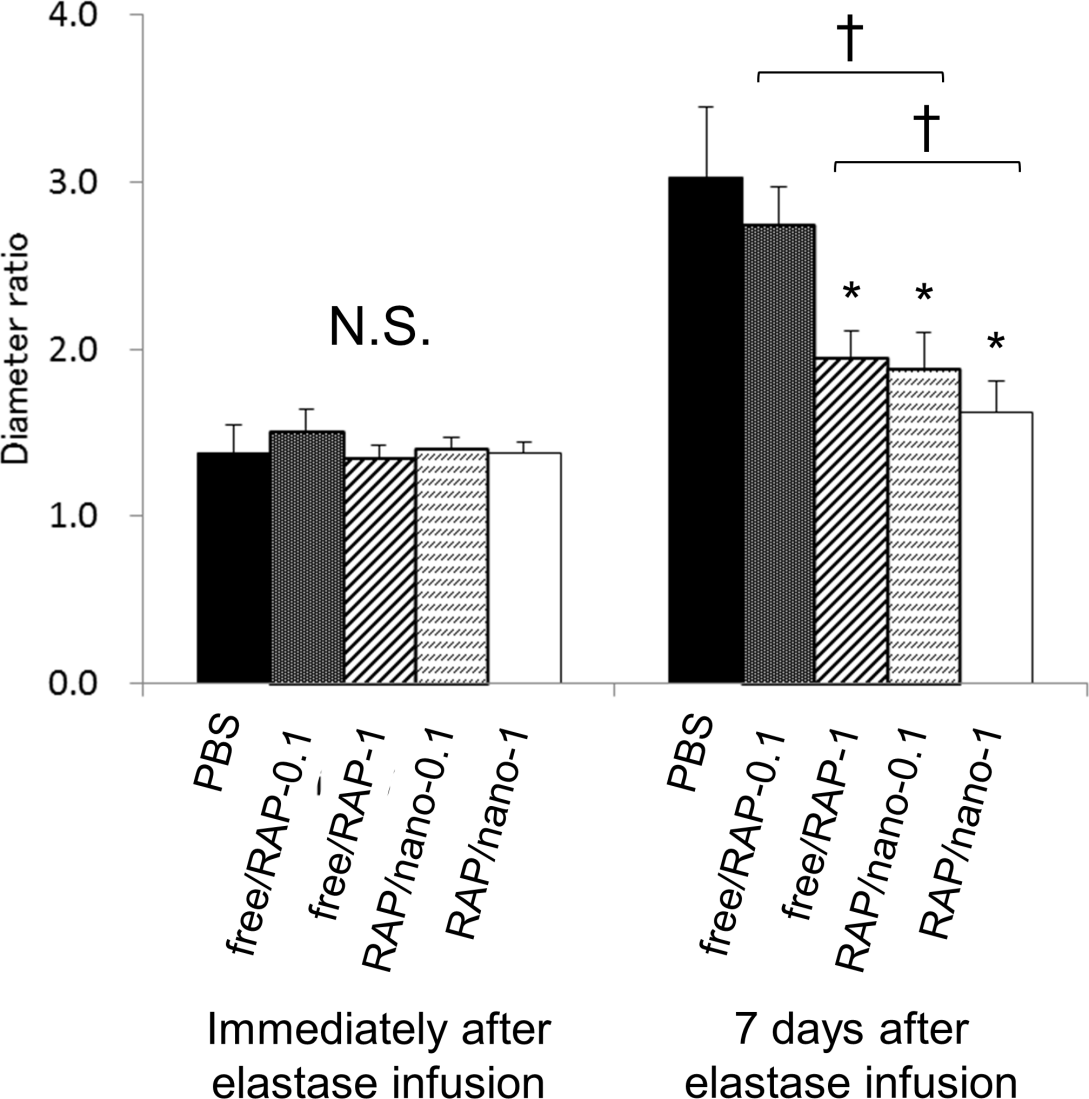
Therapeutic efficacy of rapamycin nanoparticles in the AAA rat model. The diameter of the abdominal aorta in the rat AAA model was measured before, immediately after, and 7 days after elastase infusion. The diameter ratio is expressed for each rat as the ratio of the diameter after elastase infusion to the diameter before elastase infusion. During the process of AAA formation, the rats received intravenous injections of following suspension or solvent (*n* = 6/group); i) phosphate buffered saline (PBS), ii) 0.1 mg/kg free rapamycin (free/RAP-0.1), iii) 1 mg/kg free rapamycin (free/RAP-1), iv) 0.1 mg/kg rapamycin nanoparticles (RAP/nano-0.1), or v) 1 mg/kg rapamycin nanoparticles (RAP/nano-1). RAP/nano-0.1 and RAP/nano-1 enhanced the inhibitory effect on AAA enlargement compared with free/RAP-0.1 and free/RAP-1, respectively. Data represent the means ± s.d. N.S. indicates no significant difference, *p < 0.05 (Dunnett’s test), †*p* < 0.01 (unpaired Student’s *t*-test).

During the process of AAA formation after elastase infusion, rats received intravenous injections of the following suspension or solution; i) PBS, ii) 0.1 mg/kg free rapamycin (free/RAP-0.1), iii) 1 mg/kg free rapamycin (free/RAP-1), iv) 0.1 mg/kg rapamycin nanoparticles (RAP/nano-1), or v) 1 mg/kg rapamycin nanoparticles (RAP/nano-1). All animals survived this experiment. Immediately after elastase infusion, no significant difference was detected in the sizes of the abdominal aortas between the five experimental groups (Fig 5). At 7 days after elastase infusion, the sizes of the induced AAAs after injections of free/RAP-1, RAP/nano-0.1, and RAP/nano-1 were significantly smaller than those after PBS injections (Fig 5). Furthermore, RAP/nano-0.1 and RAP/nano-1 enhanced the inhibitory effect on aneurysmal growth compared with free/RAP-0.1 and free/RAP-1, respectively (Fig 5). Blood samples were also collected from all animals at day 7, and biochemical examination of them showed no significant adverse effects after the treatment of RAP/nano-0.1 and RAP/nano-1 (S1 Table).

### Histology after treatment with rapamycin nanoparticles

**Fig 6:**
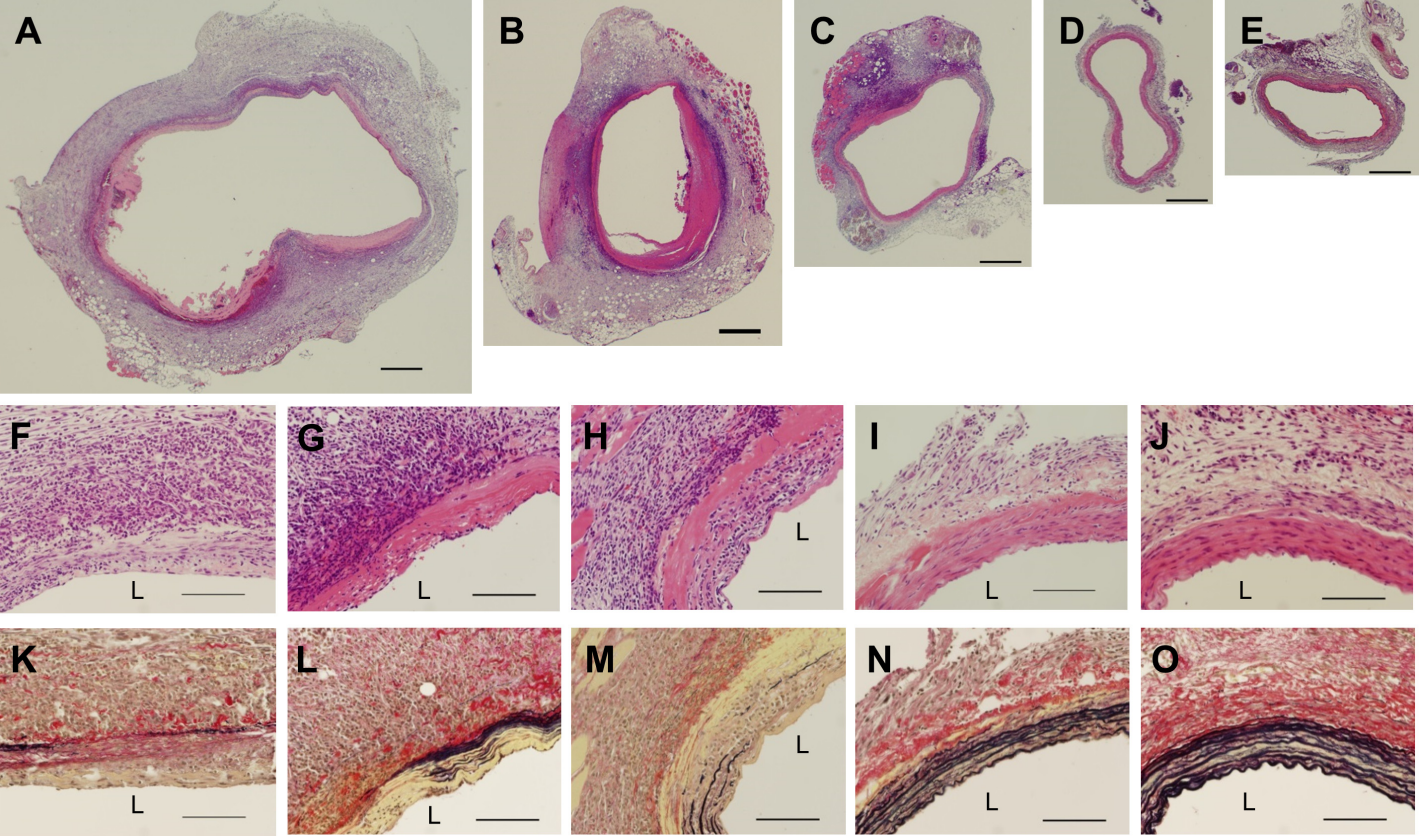
Histopathological findings of AAAs after treatment with rapamycin nanoparticles. Low power field images (A-E, scale bar = 500 μm) and high power field images (F-O, scale bar = 100 μm) of the rat AAA at 7 days after elastase infusion. The sections were stained by hematoxylin-eosin (A-J) or via the Elastica van Gieson method (K-O). During the process of AAA development, the rats received intravenous injections of PBS (A,F,K), free/RAP-0.1 (B,G,L), free/RAP-1 (C,H,M), RAP/nano-1 (D,I,N), or RAP/nano-1 (E,J,O). The size of the AAA after injections of RAP/nano-0.1 (D) and RAP/nano-1 (E) are smaller than those after the other injections (A–C). Considerable numbers of inflammatory cells are observed in the AAA after injections of PBS (F), free/RAP-0.1 (G), and free/RAP-1 (H), with concomitant destruction of the medial elastic laminae (K–M). L indicates the lumen.

The AAA samples obtained during the above experiment were histologically investigated by HE and EVG staining. In low-power fields, the cross-sectional areas of the AAAs after injections of RAP/nano-1 were smallest among those of all experimental groups, and the areas of the AAAs after injections of RAP/nano-0.1 and free/RAP-1 were slightly larger (Fig 6A–6C). In contrast, the areas of the AAAs after injections of PBS and free/RAP-0.1 were markedly enlarged as compared with those after the other injections; in addition, mural thrombi were adhered on the luminal surface of these AAAs (Fig 6D and 6E). Observation in higher-power fields revealed that the findings of the AAA wall after injections of RAP/nano-0.1 and RAP/nano-1 were considerably different from those after injections of PBS, free/RAP-0.1, and free/RAP-1. The HE stain showed little accumulation of inflammatory cells in the AAA wall after the injections of RAP/nano-0.1 and RAP/nano-1, whereas inflammatory cells abundantly accumulated after the injections of RAP/nano-0.1 and RAP/nano-1 (Fig 6F–6J). Additionally, the EVG stain showed that the structures of the medial elastic laminae were preserved after the injections of RAP/nano-0.1 and RAP/nano-1, but were remarkably destroyed after the injections of PBS, free/RAP-0.1, and free/RAP-1 (Fig 6K–6O).

**Fig 7:**
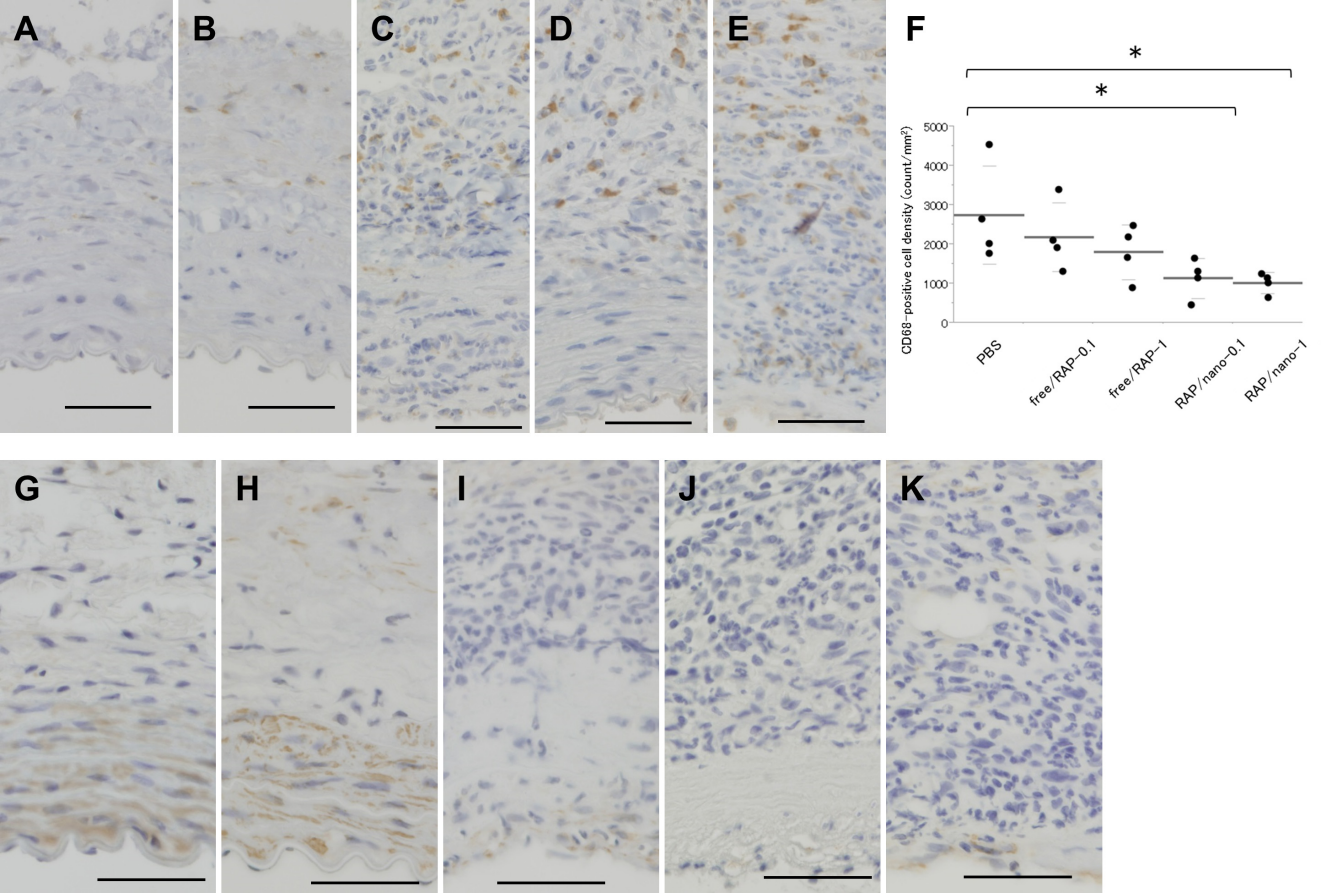
Immunohistological findings of AAA after treatment by rapamycin nanoparticles. (A–E) Micrographs of the rat AAA at 7 days after elastase infusion; the sections were immunostained for CD68 (brown). Abundant infiltrations of CD68-positive cells are observed in the AAA after injections of PBS (A), free/RAP-0.1 (B) and free/RAP-1 (C), whereas scarce after injections of RAP/nano-0.1 (D) and RAP/nano-1 (E). To quantify the density of CD68-positive cells in AAA wall, CD68-positive cell density of each section was calculated (F). Black dots represent the specific values in each group. Long and short bars represent mean and standard deviation, respectively. *p < .05. (G–K) Micrographs of AAA at 7 days after elastase infusion; the sections were stained for α-smooth muscle actin (αSMA, brown). The density of αSMA-positive cells in the media markedly decreased in the AAA after injections of PBS (G), free/RAP-0.1 (H), and free/RAP-1 (I), while that was preserved in the AAA after injections of RAP/nano-0.1 (J) and RAP/nano-1 (K). Scale bar = 50 μm. *p < 0.05 (Dunnett’s test).

The AAA samples were also assessed by immuno-staining. The immuno-stain for CD68 showed a scattering accumulation of CD68-positive cells mainly in the adventitia of all experimental groups. The numbers of CD68-positive cells after injections of RAP/nano-0.1 and RAP/nano-1 were scarce as compared with those after injections of PBS, free/RAP-0.1, and free/RAP-1 (Fig 7A–7E). Similarly, CD68-positive cell densities after the injections of RAP/nano-0.1 and RAP/nano-1 were significantly lower than that after PBS injection (Fig 7F). On the other hand, the immuno-staining for αSMA demonstrated that αSMA-positive cells in the media were preserved after injections of RAP/nano-0.1 and RAP/nano-1, whereas inversely few αSMA-positive cells were detected in the media after injections of PBS, free/RAP-0.1, and free/RAP-1 (Fig 7G–7K).

### Gelatinase activity and expression of inflammatory cytokines

Another set of rats was subjected to elastase infusion, and then they received intravenous injections of PBS, free/RAP-1, or RAP/nano-1 immediately after and at 2, 4, and 6 days after elastase infusion. At 7 days after elastase infusion, the segment of the AAA was collected from each animal and evaluated by zymography and Profiler array of protein expression.

**Fig 8:**
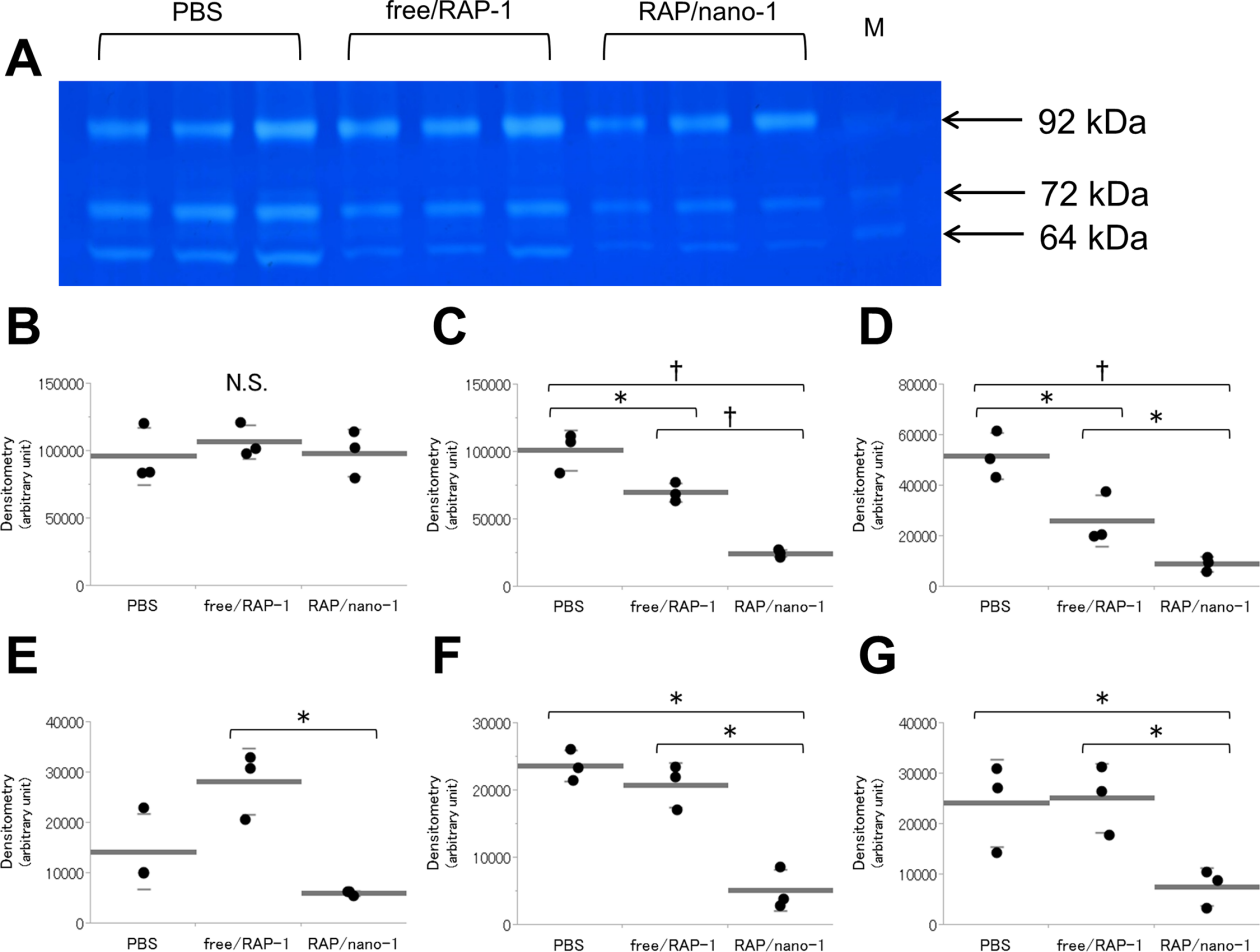
Gelatinase activities and expression of inflammation-related factors in AAA homogenates after treatment of rapamycin nanoparticles. (A) Gelatin zymography of AAA lysates. The rats were subjected to elastase infusion, and 7 days later, AAA samples were collected. During the process of AAA formation, the rats received intravenous injections of PBS (*n* = 3), free/RAP-1 (*n* = 3), or RAP/nano-1 (*n* = 3). Gelatinase activities at 64, 72, and 92 kD represent matrix metalloproteinase-2 (MMP-2), latent form MMP-2 (pro-MMP-2), and latent form MMP-9 (pro-MMP-9), respectively. The MMP marker containing MMP-2, pro-MMP-2, and pro-MMP-9 was applied in the most right lane (M). (B–D) Gelatinase activities of MMP-9 (B), pro-MMP-2 (C), and pro-MMP-2 (D) were quantified by densitometry. In the expressions of pro-MMP-2 and MMP-2, the values after injections of RAP/nano-1 were significantly reduced as compared with those after injections of PBS and free/RAP-1. Contrarily, no differences were detected in the activities of pro-MMP-9 between the 3 treatments. Profiler array analyses of AAA at 7 days after elastase infusion revealed significant findings in expressions of interleukin (IL)-1α (E), IL-1β (F), and cytokine-induced neutrophil chemoattractant (CINC)-1 (G). The expression of IL-1α, IL-1β, and CINC-1 were significantly suppressed in the AAA after injections of RAP/nano-1 as compared with those after injections of PBS and free/RAP-1 (n = 3 for each). The experiments were repeated 3 times. In panels B–G, black dots represent the specific values in each group. Long and short bars represent mean and standard deviation, respectively. Error bars denote s.d. *p < 0.05, †*p* < 0.01 (unpaired Student’s *t*-test).

Gelatin-zymography successfully displayed gelatinase activities at 64, 72, and 92 kDa, which corresponded to MMP-2, the latent form MMP-2 (pro-MMP-2), and the latent form MMP-9 (pro-MMP-9), respectively (Fig 8). The activities of MMP-2 and pro-MMP-2 after injections of RAP/nano-1 were markedly suppressed as compared with those after injections of PBS and free/RAP-1, whereas the same activities after free/RAP-1 injections were also less than those after PBS injections. In contrast, no visible differences were observed between the pro-MMP-9 activities after injections of PBS, free/RAP-1, and RAP/nano-1.

Various cytokines and chemokines related to the regional inflammatory reaction were surveyed using the Profiler array [32]. Among the series of cytokines and chemokines, significant findings were detected only for IL-1α, IL-1β, and CINC-1, and their expression after RAP/nano-1 injections was significantly down-regulated as compared with that after injections of PBS and free/RAP-1 (Fig 8).

## Discussion

In the present study, we attempted to incorporate rapamycin into nanoparticles composed of PEG-*b*-PBLG. Because PEG-*b*-PBLG is an amphiphilic block copolymer, the PEG-*b*-PBLG molecules associate in water to form a nanoparticle that possesses a unique core-shell structure of a hydrophilic PEG shell surrounding a PBLG core. Rapamycin is a hydrophobic drug [30, 33]. The flash mix of rapamycin and PEG-*b*-PBLG in DMF/water promotes the incorporation of the rapamycin molecule into the core of the PEG-shelled particle by hydrophobic interaction. Indeed, measurement with a zetasizer demonstrated that uniform particles with a diameter of 106 nm were generated after mixing rapamycin and PEG-*b*-PBLG, and the size of acquired particles was approximately 60 nm larger than that of the particles formed solely with PEG-*b*-PBLG. These findings suggested that rapamycin was successfully loaded into the nanoparticles. Incorporation of rapamycin into block copolymer-based micelles has also been reported in previous studies [34, 35]. The time-course analyses of scattered light intensity of the rapamycin nanoparticles revealed their structural stability under physiological conditions for 48 hours, indicating that the rapamycin nanoparticles could be administered via the blood stream. Moreover, previous studies reported that cytotoxicity of PEG and PBLG was low enough to be utilized in clinical settings [36–38].

Following the preparation of the rapamycin nanoparticle, we intravenously injected Alexa647-labeled rapamycin nanoparticles into the rats in which AAA had been induced, and then evaluations of aorto-iliac specimens revealed that abundant signals of Alexa647 were distributed to the AAA as compared with other parts of the specimens. Further, the distribution of Alexa647 in the AAA remained even at 24 hours after the injection. The Alexa647 findings suggested that circulating nanoparticles could specifically accumulate in the AAA wall over a considerable period of time.

One reasonable explanation for the particle accumulation in AAA might be that rapamycin nanoparticles got into the micro-defects of the disrupted aneurysmal wall and were retained in these regions. Although the pathogenesis of human aortic aneurysm is not fully understood, infiltration of inflammatory cells in the wall is considered to be a key process in the formation of aortic aneurysm [4, 35, 39, 40]. The infiltrated cells release inflammatory cytokines, leading to expression of several proteases in the aortic wall [39]. These cytokines and proteases coordinately induce degradation of structural matrix in media and adventitia and loss of medial SMCs, and these biochemical events decrease tensile strength of the aortic wall followed by aneurysmal formation [4, 41]. Reflecting the aforementioned process, conspicuous histopathological features of aortic aneurysm are marked destruction of the wall structure including fragmentation of medial elastic lamella, thinning of medial SMCs, and transmural infiltration of macrophages and lymphocytes, which are reproduced in the rat AAA induced by elastase infusion [4, 39, 42–46]. In these findings, the marked destruction of the aneurysmal wall implicates abundant existence of micro-defects in the wall structure. Further, since the endothelial layer is extensively damaged in most aortic aneurysms, nanoparticles in the blood stream might be able to get into the aneurysmal wall via the micro-defects. Histological analyses of rat AAA after injection of Alexa647-labeled rapamycin nanoparticles indeed showed that the distribution of Alexa647 in the AAA wall was notable in the parts with progressive destruction of the wall structure, and there was less distribution of Alexa647 dots in the wall with preserved structure. This contrast of Alexa647 distribution might support the above mechanism of particle accumulation. Incidentally, the size of the particles might be critical to realize effective accumulation in the aneurysmal wall by this mechanism. If the size of particles exceeds those of the micro-defects of aortic aneurysm, the particles cannot get into the aneurysmal wall. If the particle size is too small, the particles would hardly remain in the aneurysmal wall, since such small particles potentially migrate various directions via interstices of tissues. Although the present study did not examine the appropriate size of particles to achieve effective accumulation in the rat AAA, the sustained accumulation of rapamycin nanoparticles in the AAA might indicate that 106 nm of mean diameter was within the range of adequate size for particle accumulation in the aneurysmal wall.

Medial neovascularization in the aneurysmal wall might also explain the accumulation of rapamycin nanoparticles in AAA [47]. As described, inflammatory cells were extensively infiltrated in the wall of aortic aneurysm, and the inflammatory cells release several cytokines in the aneurysmal wall. Since some of such inflammatory cytokines possess potent angiogenic effects, neovascularization is considerably induced in the medial layer of aortic aneurysm [48, 49], and the supplement of this study showed similar histopathological finding in the rat AAA (S2 Fig). Generally, the structure of neovessels is immature because of inadequate lining by pericytes [48, 49], and inflammatory cytokines and proteases increase vascular permeability [50, 51]. Therefore, we suspected that part of the rapamycin nanoparticles in the blood stream might be distributed to the AAA wall through the developed neovascularization in the aneurysmal media.

Similarly, the inflammatory cells and cytokines potentially increased permeability of the vasa vasorum in the outer media and adventitia, which might enhance accumulation of nanoparticles in the AAA wall. Histological evaluation showed that Alexa647-labeled nanoparticles were considerably distributed in the adventitia of the AAA wall, also reflecting the potent role of the vasa vasorum.

On the basis of the distribution analyses of rapamycin nanoparticles, we next evaluated therapeutic effects of the rapamycin nanoparticles in the rat model of AAA. The morphological analyses at 7 days after elastase infusion clarified that intravenous administration of rapamycin nanoparticles significantly suppressed aneurysmal formation as compared with that of free rapamycin with the same dose, indicating that the delivery by the nanoparticles enhanced therapeutic efficacy of rapamycin. The enhancement of rapamycin’s effect might be attributable to the distributional property of rapamycin nanoparticles. After the accumulation of rapamycin nanoparticles in the AAA, rapamycin might have dissociated from the particles and then affected the cells in the aneurysmal wall, resulting in the specific delivery of rapamycin to the AAA. Regarding the dissociation of rapamycin from the particles, we suspect two possible processes, because the target molecule of rapamycin is an intracellular protein kinase named mammalian target of rapamycin (mTOR) [52]. One is an intracellular dissociation after endocytosis of rapamycin nanoparticles, and the other process is an extracellular dissociation followed by transportation of free rapamycin into the cells. Immuno-histological analyses after injection of Alexa647-labeled rapamycin nanoparticles showed co-localization of Alexa647 and CD68-positive cells in the wall of AAA. This finding suggested that rapamycin was potentially delivered to the macrophages in the AAA wall by the former process of rapamycin’s dissociation. Since the macrophage is one of the critical cells promoting the formation of aortic aneurysm as described [39, 40], the delivery of rapamycin to the macrophages could potentially explain the suppressive effect against formation of AAA. On the other hand, the immuno-histological analyses showed comparatively few Alexa647 dots in the extracellular space in the AAA wall. However, this finding did not necessarily negate the possibility of extracellular dissociation of rapamycin, because Alexa647-labeled nanoparticles must be degraded through the dissociation of rapamycin.

Rapamycin, a bacterial macrolide, is known to exhibit several medicinal effects, and the most prominent one is immune-suppressant activities [52, 53]. By acting on mTOR, rapamycin inhibits signal transductions for inflammatory reactions, which might suppress inflammatory infiltrations and activities of inflammatory cells in the developing aortic aneurysm [21]. Indeed, histological evaluations using AAA samples at day 7 demonstrated that the injections of rapamycin nanoparticles decreased accumulation of inflammatory cells and the number of macrophages as compared with the injections of PBS or free rapamycin. Other analyses of AAA at day 7 further showed that the treatment with rapamycin nanoparticles reduced the activities of MMP-2 and the expression of IL-1α, IL-1β, and CINC-1. MMPs are typical proteases which potently degrade a variety of ECM proteins, and previous studies suggested that MMP-9 played a central role in the formation of aortic aneurysm [4, 19, 41]. In the present study, we detected only the suppression of MMP-2 activity in the AAA at day 7, though there is a possibility that activity of MMP-9 was depressed in the AAA samples at other time points [4, 39]. Similarly, expression of inflammatory cytokines other than IL-1α, IL-1β, and CINC-1 was possibly suppressed at other time points [4, 39]. In any case, these anti-inflammatory effects in the AAA might prevent degradation of the wall structure and loss of medial SMCs, which was confirmed by the preservation of medial elastic lamina and SMCs. We believe that these effects by rapamycin coordinately inhibited the formation of AAA. For the future application of nanoparticles to treat aortic aneurysms in humans, other anti-inflammatory drugs with minimal side effects should also be investigated.

A limitation of this study was that the analysis after treatment was conducted on day 7 after elastase infusion. An observation in the later phase would be valuable especially considering the clinical relevance of this novel drug delivery system. Another limitation was the lack of data on the correlation between the dose of rapamycin nanoparticles and the release of rapamycin in the aortic aneurysm wall. Therefore, the actual delivery of rapamycin (not in the form of nanoparticles) to the sites of AAA is unknown.

In conclusion, we mixed rapamycin and PEG-*b*-PBLG to generate uniform nanoparticles incorporating rapamycin (rapamycin nanoparticle) which were stable under physiological conditions. When the fluorescence-labeled rapamycin nanoparticles were intravenously injected into the rat in which AAA had been induced by elastase infusion, specific and sustained accumulation of rapamycin nanoparticles was observed in the AAA wall. When the rapamycin nanoparticles were injected into the rat model during the development of AAA, the formation of AAA was significantly suppressed as compared with control animals treated with PBS or free rapamycin. The administration of rapamycin nanoparticles also decreased infiltration of macrophages, activity of MMP-2, and expression of inflammatory cytokines in the AAA wall, supporting suppressive effects of AAA formation. These findings suggest that the nanoparticle-based delivery system of the present study markedly enhanced the therapeutic effect of rapamycin for the rat AAA. A nanoparticle-based drug delivery system could potentially be used to establish an effective drug therapy targeting aortic aneurysm.

## Supporting Information

S1 Fig Quantitative analysis of co-localization of fluorescent rapamycin nanoparticles with CD68 and αSMA. (A) Co-localization analysis of rapamycin nanoparticles and CD68-positive cells in the rat AAA 7 days post induction and 24 hours after injection of Alexa647-labeled nanoparticles. Scattergram (left panel) was acquired from the original image of Fig 4B, immune-stained for CD68 (right panel). The horizontal axis and vertical axis of the scattergram represent the channels for CD68 and Alexa647, respectively. In the negative control sample for CD68 (the primary antibody omitted), the highest value of the horizontal axis was determined as the cutoff value for CD68 (arrow). Similarly, in the negative control sample for Alexa647 (Alexa647-labeled rapamycin nanoparticles not injected), the highest value of the vertical axis was determined as the cutoff value for Alexa647 (arrow head). The pixels in the area 2 in the scattergram show the Alexa647, which did not co-localize with CD68 (97 pixels), and the pixels in the area 3 represent the co-localization of Alexa647 and CD68 (75449 pixels). The rate of co-localization of Alexa647 with CD68 was calculated to be 99.9%. (B) Co-localization analysis of rapamycin nanoparticles and αSMA-positive cells in the rat AAA. Scattergram (left panel) was acquired from the original image of Fig 4C, immune-stained for αSMA (right panel). Analysis was conducted in the same manner, and the rate of co-localization of Alexa647 with αSMA was 9.3%.
(DOCX)

S2 Fig Immunostaining of the rat AAA 7 days after elastase infusion. (A) Micrographs of the rat AAA at 7 days after elastase infusion; the sections were immunostained for CD31 (brown). Microvasculatures in the media and adventitia of the AAA wall are conspicuously observed (arrows). Scale bar, 100 μm. (B) The thin wall and incompetent lining by pericytes indicate the immature nature of the micro vessels. Scale bar shows 20 μm.
(DOCX)

S1 Table Biochemical examination of blood after injection of rapamycin nanoparticles. AST, aspartate aminotransferase; ALT, alanine aminotransferase; BUN, blood urea nitrogen; Cre, creatinine; * *P* < 0.05.
(DOCX)
