# Supplementary material for: Nanoparticles Effectively Target Rapamycin Delivery to Sites of Experimental Aortic Aneurysm in Rats
Source: PLoS One. 2016 Jun 23;11(6):e0157813. doi: 10.1371/journal.pone.0157813 (PMC4919101; doi:10.1371/journal.pone.0157813)
Supplement: S1 Fig — (A) Co-localization analysis of rapamycin nanoparticles and CD68-positive cells in the rat AAA 7 days post induction and 24 hours after injection of Alexa647-labeled nanoparticles. Scattergram (left panel) was acquired from the original image of Fig 4B, immune-stained for CD68 (right panel). The horizontal axis and vertical axis of the scattergram represent the channels for CD68 and Alexa647, respectively. In the negative control sample for CD68 (the primary antibody omitted), the highest value of the horizontal axis was determined as the cutoff value for CD68 (arrow). Similarly, in the negative control sample for Alexa647 (Alexa647-labeled rapamycin nanoparticles not injected), the highest value of the vertical axis was determined as the cutoff value for Alexa647 (arrow head). The pixels in the area 2 in the scattergram show the Alexa647, which did not co-localize with CD68 (97 pixels), and the pixels in the area 3 represent the co-localization of Alexa647 and CD68 (75449 pixels). The rate of co-localization of Alexa647 with CD68 was calculated to be 99.9%. (B) Co-localization analysis of rapamycin nanoparticles and αSMA-positive cells in the rat AAA. Scattergram (left panel) was acquired from the original image of Fig 4C, immune-stained for αSMA (right panel). Analysis was conducted in the same manner, and the rate of co-localization of Alexa647 with αSMA was 9.3%. (DOCX) [file pone.0157813.s001.docx]

**S1 Fig. Quantitative analysis of co-localization of fluorescent rapamycin nanoparticles with CD68 and αSMA**


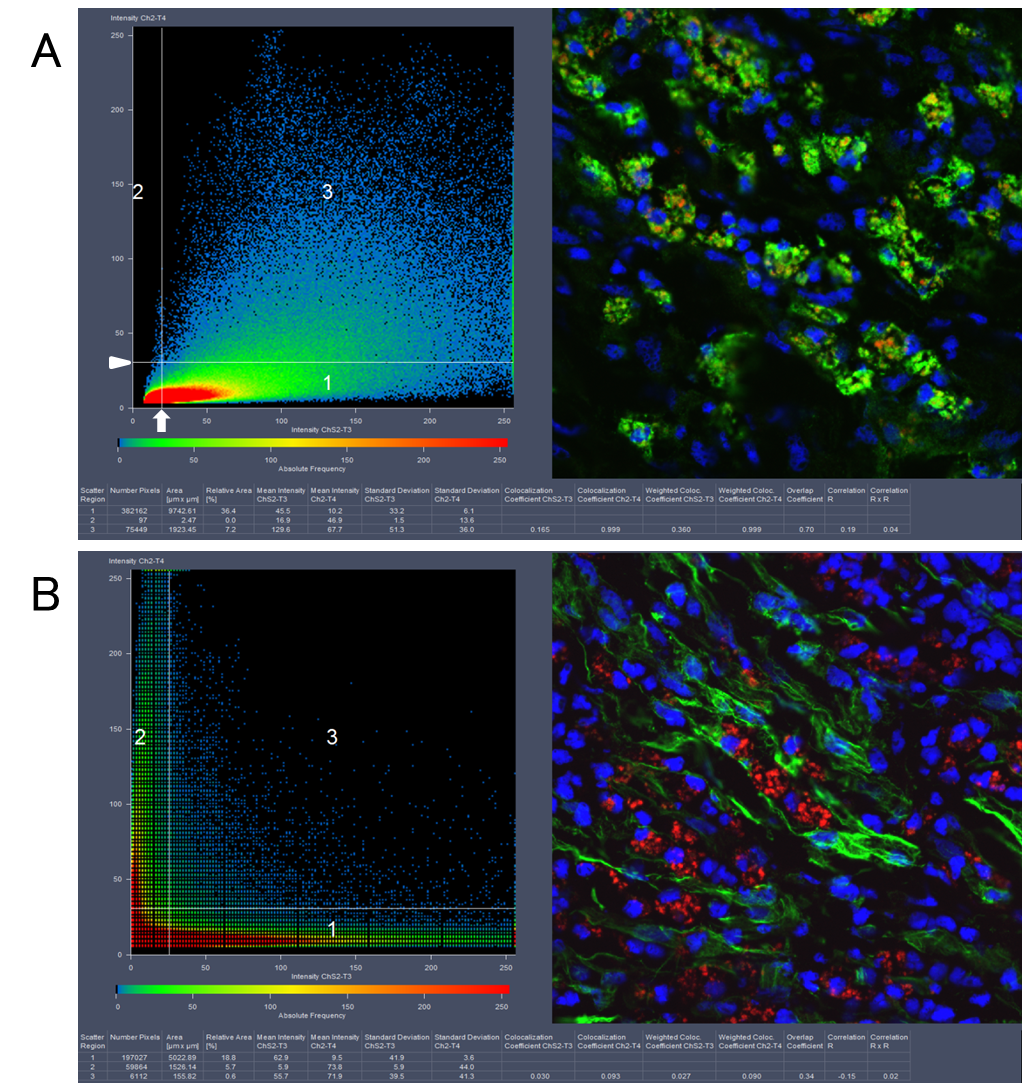


(A) Co-localization analysis of rapamycin nanoparticles and CD68-positive cells in the rat AAA 7 days post induction and 24 hours after injection of Alexa647-labeled nanoparticles. Scattergram (left panel) was acquired from the original image of Fig. 4B, immune-stained for CD68 (right panel). The horizontal axis and vertical axis of the scattergram represent the channels for CD68 and Alexa647, respectively. In the negative control sample for CD68 (the primary antibody omitted), the highest value of the horizontal axis was determined as the cutoff value for CD68 (arrow). Similarly, in the negative control sample for Alexa647 (Alexa647-labeled rapamycin nanoparticles not injected), the highest value of the vertical axis was determined as the cutoff value for Alexa647 (arrow head). The pixels in area 2 in the scattergram show the Alexa647 that did not co-localize with CD68 (97 pixels), and the pixels in the area 3 represent the co-localization of Alexa647 and CD68 (75449 pixels). Then, the rate of co-localization of Alexa647 with CD68 was calculated to be 99.9 %. (B) Co-localization analysis of rapamycin nanoparticles and αSMA-positive cells in the rat AAA. Scattergram (left panel) was acquired from the original image of Fig. 4C, immuno-stained for αSMA (right panel). Analysis was conducted in the same manner, and the rate of co-localization of Alexa647 with αSMA was 9.3 %.

*Medial neovascularization in the rat AAA*

The paraffin sections of rat AAA induced by elastase infusion were immune-stained for CD31 to detect neovascularization in the AAA wall. After treatment with antigen retrieval solution (Dako), hydrogen peroxide, and blocking solution (Dako), the sections were incubated with anti-CD31 monoclonal antibody (1:100, Fitzgerald Industries International, Acton, MA) overnight. Subsequently, the sections were treated with biotin-labeled secondary antibody (1:100; B-2001, Vector Laboratories) and visualized using an avidin-biotin complex method kit (ABC Elite kit, Vector).

CD31 immunostaining revealed microvasculature in the media and adventitia of the AAA wall whose structure was disrupted (S Fig. 1A, B).
