# Supplementary material for: Nanoparticles Effectively Target Rapamycin Delivery to Sites of Experimental Aortic Aneurysm in Rats
Source: PLoS One. 2016 Jun 23;11(6):e0157813. doi: 10.1371/journal.pone.0157813 (PMC4919101; doi:10.1371/journal.pone.0157813)
Supplement: S2 Fig — (A) Micrographs of the rat AAA at 7 days after elastase infusion; the sections were immunostained for CD31 (brown). Microvasculatures in the media and adventitia of the AAA wall are conspicuously observed (arrows). Scale bar, 100 μm. (B) The thin wall and incompetent lining by pericytes indicate the immature nature of the micro vessels. Scale bar shows 20 μm. (DOCX) [file pone.0157813.s002.docx]

**S2 Fig. Immunostaining of the rat AAA 7 days after elastase infusion**


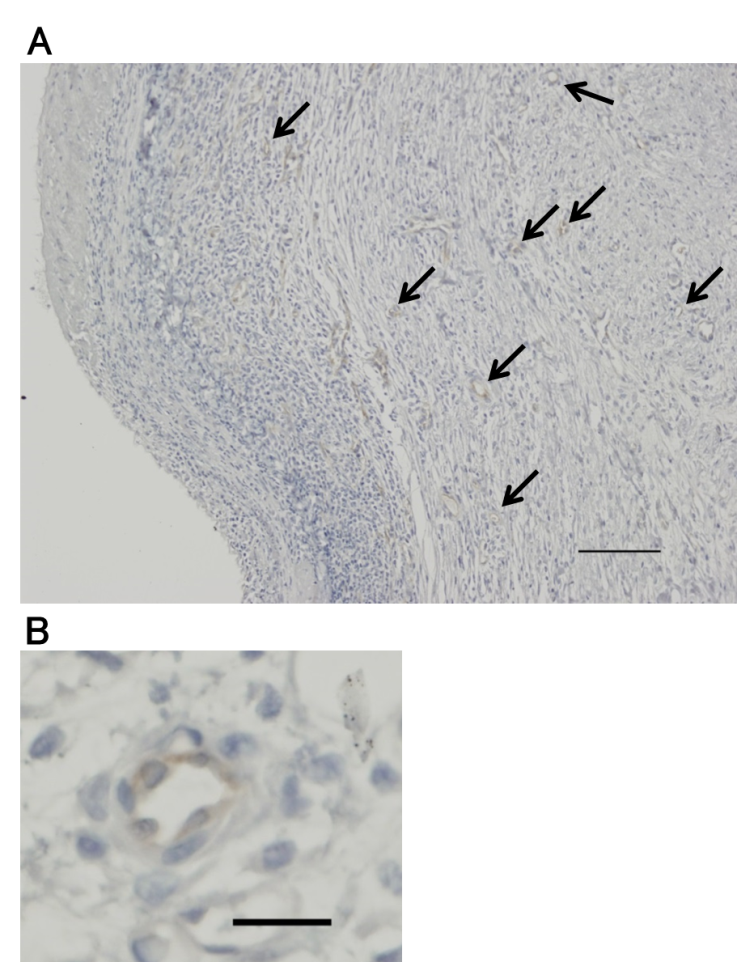


(A) Micrographs of the rat AAA at 7 days after elastase infusion; the sections were immunostained for CD31 (brown). Microvasculatures in the media and adventitia of the AAA wall are conspicuously observed (arrows). Scale bar, 100 μm. (B) The thin wall and incompetent lining by pericytes indicate the immature nature of the micro vessels. Scale bar is 20 μm.
