# Supplementary material for: Nanoparticles Effectively Target Rapamycin Delivery to Sites of Experimental Aortic Aneurysm in Rats
Source: PLoS One. 2016 Jun 23;11(6):e0157813. doi: 10.1371/journal.pone.0157813 (PMC4919101; doi:10.1371/journal.pone.0157813)
Supplement: S1 Table — AST, aspartate aminotransferase; ALT, alanine aminotransferase; BUN, blood urea nitrogen; Cre, creatinine; * P < 0.05. (DOCX) [file pone.0157813.s003.docx]

**[Supplement](http://ejje.weblio.jp/content/supplement)**

*Biochemical examination of blood after injection of rapamycin nanoparticles*

In the experiment to evaluate therapeutic effects of rapamycin nanoparticles in the model of rat AAA, blood samples were collected from all animals immediately after their sacrifice. From the blood samples, serum was separated, and aspartate aminotransferase, alanine aminotransferase, blood urea nitrogen and creatinine in the serum were measured (SRL, Tokyo, Japan).

The biochemical examination of blood detected slight increase of blood urea nitrogen only after injections of free/RAP-1 (S1 Table).

**S1 Table. Biochemical examination of blood after injection of rapamycin nanoparticles**

|  | PBS | free/RAP-0.1 | free/RAP-1 | RAP/nano-0.1 | RAP/nano-1 |
| --- | --- | --- | --- | --- | --- |
| AST, IU/L | 61±8 | 71±15 | 63±16 | 72±5 | 61±10 |
| ALT, IU/L | 23±5 | 26±4 | 24±4 | 25±4 | 25±4 |
| BUN, mg/dL | 20.8±2.4 | 21.7±4.2 | 29.0±2.3* | 22.3±3.7 | 22.6±56 |
| Cre, mg/dL | 0.4±0.2 | 0.3±0.1 | 0.2±0.2 | 0.2±0.1 | 0.2±0.1 |

AST, aspartate aminotransferase; ALT, alanine aminotransferase; BUN, blood urea nitrogen; Cre, creatinine; * *p* < 0.05
